# Reduction of Exciton Diffusion Length with Genetically Tuned Non-Photochemical Quenching in Plant Thylakoid Membranes

**DOI:** 10.1021/acs.jpclett.5c01473

**Published:** 2025-07-14

**Authors:** Tsung-Yen Lee, Lam Lam, Dhruv Patel-Tupper, Henry E. Lam, Krishna K. Niyogi, Graham R. Fleming

**Affiliations:** † Department of Chemistry, 1438 University of California, Berkeley, California 94720, United States; ‡ Molecular Biophysics and Integrated Bioimaging Division, Lawrence Berkeley National Laboratory, Berkeley, California 94720, United States; § Graduate Group in Biophysics, University of California, Berkeley, California 94720, United States; ∥ Department of Plant and Microbial Biology, University of California, Berkeley, California 94720, United States; ⊥ Howard Hughes Medical Institute, University of California, Berkeley, California 94720, United States; # Innovative Genomics Institute, University of California, Berkeley, California 94720, United States; 7 Kavli Energy Nanoscience Institute at Berkeley, Berkeley, California 94720, United States

## Abstract

Non-photochemical quenching (NPQ) protects plants from excess light by dissipating excitation energy as heat. Limited exciton migration is a key feature of NPQ, reducing the energy flux to reaction centers; however, quantitative evidence for this mechanism in native thylakoid membranes has been lacking. Here, we investigate the correlation between NPQ activity and exciton diffusion length (*L*_D_) using *Nicotiana benthamiana* mutants with distinct NPQ capacities. NPQ under both light- and dark-acclimated conditions was quantified for each genotype via fluorescence lifetime snapshots, and exciton mobility was probed using transient absorption spectroscopy with exciton–exciton annihilation analysis. By comparing the relationship between chlorophyll fluorescence lifetime and *L*_D_ across mutants, we observed that NPQ activation quantitatively limits the spatial range of exciton migration, thereby reducing the access to reaction centers. Our findings provide direct experimental evidence that NPQ modulates the dynamics of energy transport, advancing our understanding of photoprotective regulation in photosynthetic systems.

## Graphical abstract

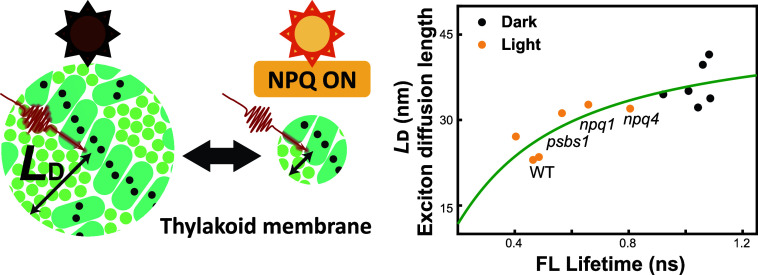

Photosynthesis begins with light absorption by chlorophyll (Chl) in light harvesting complex II-photosystem II supercomplexes (LHCII-PSII). In grana membranes, the antenna complexes and supercomplexes form a compact membrane structure, allowing excitons to travel within the Chl network and ultimately reach the reaction center in PSII, where charge separation occurs.^1–4^ The exciton mobility determines the photosynthetic efficiency and the regulation of photoprotection by controlling the time scales for encountering reaction centers (RCs) or energy quenchers.^5^

Non-photochemical quenching (NPQ) plays a crucial role in protecting photosynthetic organisms from photodamage by safely dissipating excess light energy as heat.^6–8^ The largest component of NPQ is referred to as energy-dependent quenching (qE), which is a rapidly reversible response to fluctuating light intensity.^9–11^ The activation of qE is a pH-dependent process involving a xanthophyll conversion known as the VAZ cycle.^12,13^ Following high light induced lumen acidification, Violaxanthin (Vio) is enzymatically converted into Zeaxanthin (Zea), one of the primary energy quenchers.^14–17^ Additionally, the pH sensing protein PsbS responds to the transmembrane proton gradient, putatively converting the light-harvesting complex II (LHCII) into an active quenching configuration via yet unresolved mechanisms.^18–20^

The exciton diffusion length (*L*_D_) is a key parameter for understanding exciton mobility in energy transfer networks. However, accurately measuring *L*_D_ *in vivo* remains challenging due to the structural heterogeneity of the thylakoid membrane, its functional dependence on an intact chloroplastic environment, and the small size of membrane domains, all of which complicate direct measurements. Exciton diffusion properties have been studied in isolated LHCII complexes,^21–23^ but similar investigations in intact thylakoids remain scarce. This has led to variable and inconsistent fluorescence yield-based estimates, with exciton diffusion coefficients of 1 to 10 × 10^–3^ cm^2^/s and roughly estimated *L*_D_ of 20–80 nm in thylakoid membranes.^24^

Bennett et al. employed multiscale models to simulate exciton migration in a membrane array with a tunable NPQ quencher activity, estimating an *L*_D_ of ∼50 nm under dark conditions.^25^ Notably, this model suggested that *L*_D_ is the key factor in describing the influence of qE on photosynthetic performance. The effect of NPQ can be quantitatively represented by the correlation between *L*_D_ and photochemical yield. Under excess light, activated quenchers act as weak energy traps, reducing overall exciton mobility, shrinking the *L*_D_, and consequently decreasing the energy flux to the RCs. This mechanism prevents photodamage from excess light energy as a result of closed RCs and consequent formation of reactive oxygen species.^26,27^ Bennett et al. predicted an inverse relationship between NPQ and *L*_D_. However, direct experimental validation of this relationship remains limited, with only preliminary evidence from our previous work.^17^

In this study, we experimentally explore the correlation between NPQ and *L*_D_ in *Nicotiana benthamiana* thylakoid membranes. To investigate how different NPQ capacities affect *L*_D_, we examine wild-type (WT) plants along with various NPQ-related mutants (*zep2*, *lut2*, *psbs1*, *npq1*, and *npq4*), each defective in specific qE-related pigments or proteins. The *zep2* mutant lacks one of the two zeaxanthin epoxidase paralogs, leading to constitutive Zea accumulation by blocking the reverse pathway of the VAZ cycle, while retaining near-native compositions of other photosynthetic xanthophylls, such as Vio and Neoxanthin.^28,29^ Conversely, the *npq1* mutant is deficient in violaxanthin de-epoxidase, preventing the formation of Zea in the VAZ cycle.^30^ The *lut2* mutant has lost the ability to synthesize lutein (Lut), a potential qE quencher^31^ and a structural molecule critical for LHCII trimeric stability.^17,32^ *npq4* lacks the pH sensing protein, PsbS, which induces protein conformational changes that activate bound Zea or Lut in LHCII as active quenchers.^9^ In contrast, *psbs1* lacks the dominant copy of two PsbS-encoding homologues, reducing PsbS abundance in thylakoid membranes and overall NPQ capacity.^17,33^

To investigate the exciton diffusion behavior in *N. benthamiana* thylakoid membranes with various NPQ capacities, we first determine NPQ activation levels using the fluorescence lifetime snapshot technique in alternating light-dark sequences. Fluorescence lifetime measurement provides the quantum yield of Chl fluorescence and allows us to estimate energy quenching in response to high light, independent of the size of the antenna systems or light absorbance which can bias traditional pulse-amplitude modulated fluorometry measurements. The quenching of fluorescence lifetime is quantified as NPQ_τ_, which effectively measures a plant’s NPQ response after high light exposure (relative to the dark-acclimated state). However, pleiotropic variation across mutants with different xanthophyll compositions likely alters their baseline, dark-acclimated “non-quenched” states, making NPQ_τ_ less reliable for comparing the absolute extent of quenching between genotypes. Thus, direct analysis of fluorescence lifetimes provides a more reliable estimate of the quenching.

Next, we use exciton–exciton annihilation (EEA) dynamics, a process that strongly depends on exciton motion, as a probe for exciton migration. EEA occurs when two excitons travel through the system and interact upon reaching the reaction radius. One exciton transfers the energy to the other and decays nonradiatively to its ground state. The second exciton is excited to a two-exciton state, followed by a rapid relaxation to a one-exciton state. We capture this two-particle dynamics by performing intensity-cycling transient absorption (TA) spectroscopy.^17,34^ The rising component of the isolated fifth-order nonlinear signal indicates the EEA rate. Finally, we apply a diffusion model to extract the diffusion coefficient and evaluate *L*_D_, further examining its relationship with NPQ activity across genotypes.

qE activates the quenching sites that dissipate excess excitation energy through a nonradiative relaxation pathway, additional to the intrinsic ^1^Chl* fluorescence. This process leads to a reduction in the fluorescence lifetime (τ_F_) of ^1^Chl*, which can be described using the Stern–Volmer relationship:^35^

1 $$\frac{1}{\tau_{F}}=\frac{1}{\tau_{F0}}+k_{NPQ}$$

where τ_F0_ represents the intrinsic ^1^Chl* fluorescence lifetime resulting from the radiative and nonradiative relaxation pathways. *k*_NPQ_ is the NPQ quenching decay rate, resulting in τ_F_ reduction when activated under excess light. Time-correlated single photon counting (TCSPC) is widely used to measure τ_F_ and quantify the extent of NPQ, denoted as NPQ_τ_. To capture the NPQ response to the light and dark environment, we applied a snapshot fluorescence lifetime technique where the thylakoid membrane samples are exposed to a sequence of alternating dark and light periods.^36,37^ NPQ_τ_ is defined as ${\mathrm{NPQ}}_{\tau }\left(T\right)=\frac{\tau_{F,dark}-\tau_{F,light}\left(T\right)}{\tau_{F,light}\left(T\right)}$ , where τ_F,dark_ is the amplitude-weighted averaged lifetime in dark-acclimated conditions before light exposure, and τ_F,light_ is the averaged lifetime after *T* minutes of the sequence.

Figure 1 shows NPQ_τ_ snapshots for thylakoid membranes of each genotype under a 15-5-5-5 light-dark-light-dark sequence, at a light intensity of 1000 μmol photons m^–2^ s^–1^_._ In all genotypes except *npq4* mutants, NPQ_τ_ shows a rapid decrease within the first 2 min of the dark period, suggesting the rapid deactivation of qE. In contrast, *npq4* mutants exhibit a slow, continuous rise in NPQ_τ_ due to the absence of qE activity; instead, longer-acting forms of quenching such as qZ and qI likely contribute. The slight increase of NPQ_τ_ instead of recovery during the dark period is most likely due to a reaction center dependent form of photoinhibition.^38^ Interestingly, *npq1* also shows a slight NPQ_τ_ increase during the dark period. This may reflect that the absence of Zea, a key qE quencher, may cause insufficient photoprotection, leading to additional qI activation, but further work will be required to confirm the mechanism. Overall, isolated thylakoid membranes retain NPQ activity, although their NPQ induction is lower than the intact leaves with a maximum NPQ_τ_ less than 2.^39,40^

**Figure 1:**
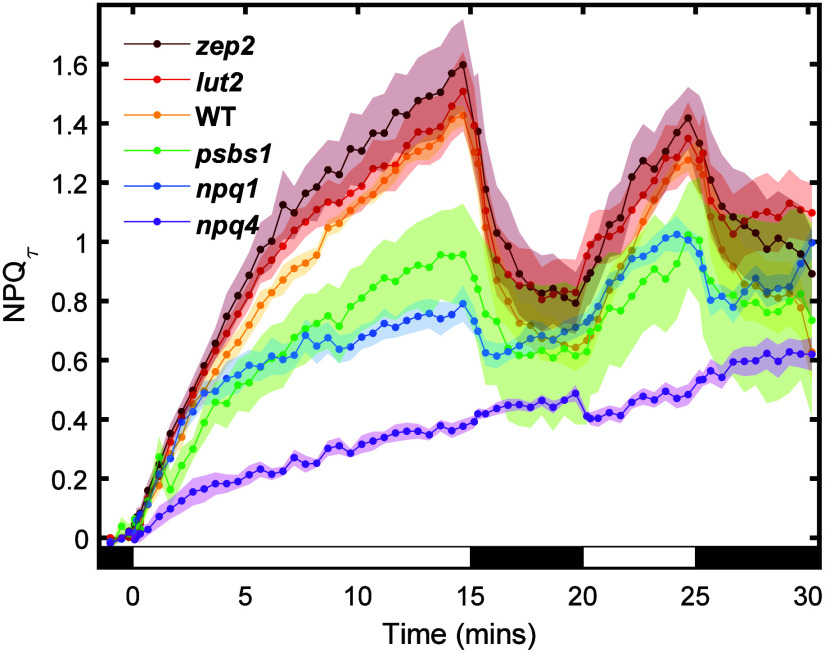
NPQ_τ_ evolution for the genotypes of the *N. benthamiana* thylakoid membranes measured by fluorescence lifetime snapshots under a 15-5-5-5 light-dark illumination sequence. The shaded areas represent the error bar, indicating the mean ± standard error (SE, *n* = 5; *n* = 4 for *psbs1*).

Because the TA measurements are performed after a period of light exposure or under dark conditions, it is necessary to define the light-acclimated (LA) and dark-acclimated (DA) NPQ_τ_ values to characterize the NPQ activity under these conditions. The LA NPQ_τ_ is obtained by averaging the NPQ_τ_ for each genotype sample between 13 and 15 min of the light sequence, representing the maximum magnitude of NPQ during the TA measurements after 15 min light exposure. The LA NPQ_τ_ values are reported in Table 1, with a rank order of *zep2* > *lut2* > WT > *psbs1* > *npq1* > *npq4*.

**Table 1:** Fluorescence Lifetime and NPQ_τ_ Results of Each Thylakoid Membrane Samples before Actinic Light Exposure (*τ*_F,dark_, −1 to 0 min in Figure 1) and under the Dark-Acclimated (DA) and Light-Acclimated (LA) Conditions^a^

	averaged TCSPC lifetime τ_F_/ns			NPQ_τ_	
genotype	τ_F,dark_	DA	LA	DA	LA
*zep2*	1.02 ± 0.05	0.92 ± 0.05	0.40 ± 0.01	0.12 ± 0.03	1.56 ± 0.15
*lut2*	1.13 ± 0.01	1.04 ± 0.08	0.46 ± 0.12	0.08 ± 0.02	1.45 ± 0.12
WT	1.16 ± 0.01	1.09 ± 0.02	0.48 ± 0.04	0.07 ± 0.02	1.40 ± 0.04
*psbs1*	1.10 ± 0.03	1.01 ± 0.04	0.57 ± 0.16	0.11 ± 0.04	0.95 ± 0.16
*npq1*	1.16 ± 0.01	1.06 ± 0.02	0.66 ± 0.05	0.10 ± 0.02	0.76 ± 0.05
*npq4*	1.10 ± 0.01	1.08 ± 0.03	0.81 ± 0.02	0.02 ± 0.03	0.37 ± 0.02

a Data are presented as means ± SE (*n* = 5; *n* = 4 for *psbs1*).

According to the definition, NPQ_τ_ values before light exposure are zero across all measured genotypes. Consequently, these values are not informative for correlating NPQ_τ_ with other variables under dark conditions. Instead, we define DA NPQ_τ_ as the averaged NPQ_τ_ within the first minute of light exposure, before significant qE has been induced, serving as an estimate of the NPQ activity during TA measurements under dark conditions. This approach is based on the assumption that the baseline laser excitation during TA induces minimal NPQ activation, with its extent roughly similar to the NPQ level observed during the first minute of actinic light exposure in TCSPC measurements.

The exciton diffusion length (*L*_D_) is obtained by probing the exciton–exciton annihilation (EEA) dynamics using intensity-cycling transient absorption (TA) spectroscopy.^34^ In our TA measurements, the signal was monitored at 680 nm, a wavelength dominated by the ground-state bleach of Chl, which provides a direct probe of the Chl population kinetics including the EEA process. TA measurements at three prescribed intensities provide third (PP3), fifth (PP5), and seventh (PP7) order nonlinear signals, corresponding to single-, two-, and three-exciton dynamics, respectively. The separation of the TA profiles into these three contributions is described as eq 4 in the final paragraphs of the main text. Figure 2 shows the third- and fifth-order signals, using *zep2* thylakoid membranes as a representative example, along with the fits using a kinetic model described in the Supporting Methods. Full TA results for all genotypes are presented in Figure S1. The decay time (τ) of the third-order nonlinear TA profile represents the exciton lifetime. In the extracted fifth-order signal, the same exciton decay kinetics is also observed with an additional rise time (τ_A_). The rising component represents the EEA rate and can be further analyzed by a diffusion model to obtain diffusion-related properties.

**Figure 2:**
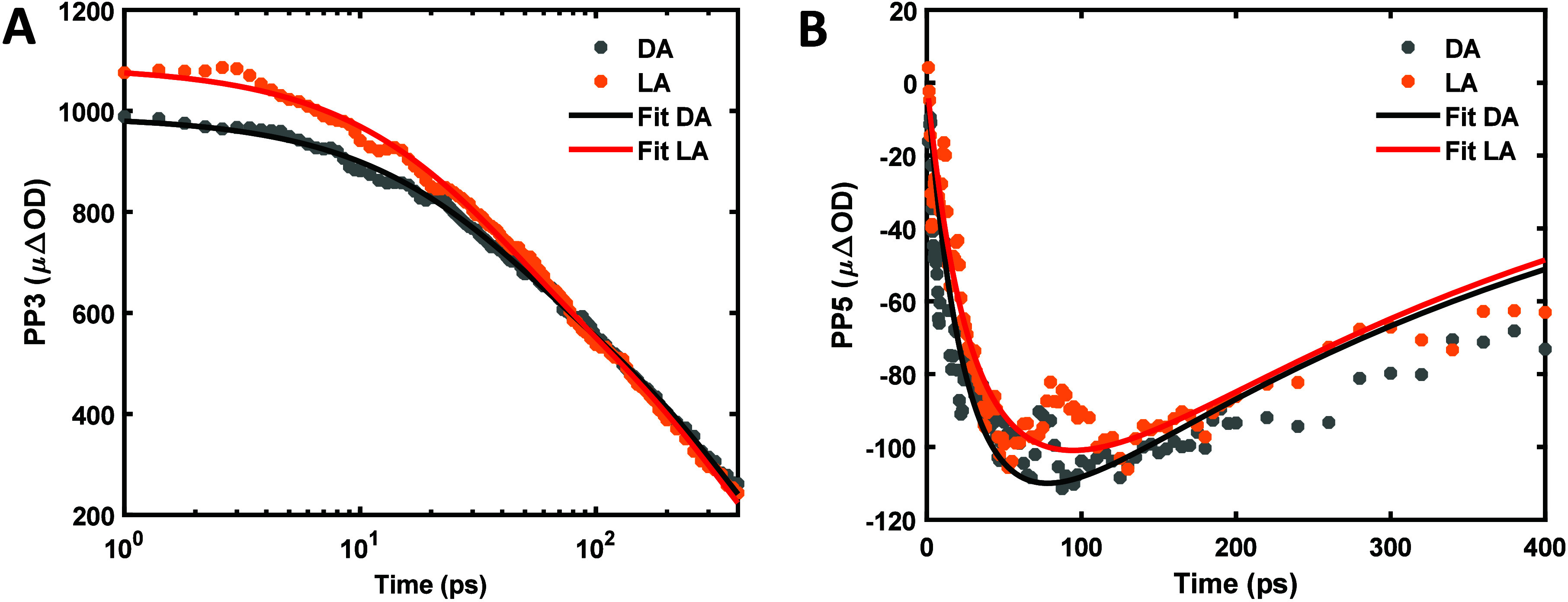
Extracted (A) PP3, (B) PP5 results, and corresponding fits for the *zep2* thylakoid membranes samples.

To analyze the EEA rate, we made the following assumptions: (1) The exciton transport in the thylakoid membranes is a three-dimensional diffusion process. (2) EEA dynamics is a diffusion-limited reaction describing the hard-sphere collisions between two exciton quasiparticles.^41,42^ (3) The spatial dimension of a two-exciton system is defined by the mean distance between Chls, where excitons can effectively encounter each other before decaying to the ground state. With these assumptions, we can evaluate the diffusion coefficient by applying the diffusion model:

2 $$1/\tau_{A}=4\pi \left(D_{m}+D_{n}\right)R\times \left(\left[S_{1}\right]/V\right)$$

where *D*_m_,*D*_n_ are the exciton diffusion coefficients of the two excitons involved in the annihilation event, labeled as m and n. *R* is the annihilation radius, set as 2 nm as the approximate mean distance between Chl domains.^24,25^ *V* represents the volume of a two-exciton system, the system size, in which two excitons can effectively encounter. *V* is a unit conversion term that converts the right side of eq 2 to SI units. [S_1_] represents the concentration of excitons and equals 2 per two-exciton system. The diffusion length (*L*_D_) is defined by

3 $$L_{D}=\sqrt{6D\tau }$$

where τ is the lifetime of excitons without EEA events, obtained from the averaged decay time of the third-order nonlinear TA profiles. We note that eq 2 shares the same form as used in ref 17 but incorporates the relative diffusion coefficient and exciton concentration for a more realistic description.

When evaluating *L*_D_ values across different genotypes, we made the following biological assumptions for simplicity: (1) The Chl energy transfer network is consistent across all genotypes, allowing us to implement the same system size and *R* values for all samples. (2) *L*_D_ represents a mean value for overall behavior of Chl excitons in the membrane due to the heterogeneity in energy transfer rates caused by the variance in interpigment and interprotein distances.^25,43^

TA measurements for each genotype were conducted under two conditions: DA conditions, where samples stay in the dark, and LA, where the thylakoid membrane samples are exposed to high light for 15 min before measurement. The extracted third- and fifth-order nonlinear TA profiles for all genotypes are shown in Figure S1. The fitted PP3 decay lifetime τ, PP5 rise time τ_A_, and the corresponding *L*_D_ values are summarized in Table 2.

**Table 2:** Fitted Lifetimes of the PP3 and PP5 Profiles, and the Estimated Diffusion Lengths *L*_D_ for Each Genotype in Thylakoid Membranes under the Dark-Acclimated (DA) and Light-Acclimated (LA) Conditions^a^

	PP3 τ/ps		PP5 τ_A_/ps		***L***_D_/nm	
genotype	DA	LA	DA	LA	DA	LA
*zep2*	280 ± 10	240 ± 10	57 ± 2	54 ± 3	35 ± 1	27 ± 1
*lut2*	250 ± 20	240 ± 10	40 ± 4	77 ± 8	32 ± 2	23 ± 1
WT	386 ± 40	320 ± 30	70 ± 3	99 ± 8	34 ± 2	24 ± 2
*psbs1*	420 ± 70	400 ± 60	60 ± 10	70 ± 10	35 ± 4	31 ± 4
*npq1*	290 ± 20	300 ± 10	31 ± 1	48 ± 2	40 ± 1	33 ± 1
*npq4*	260 ± 10	240 ± 10	26 ± 2	40 ± 2	42 ± 1	32 ± 1

a The DA results are the averaged values of the first 1 min of light exposure. Errors denote standard errors from nonlinear least-squares fits of the TA signals.

Under excess light, the activated NPQ quenchers capture excitons in the thylakoid membranes, thereby reducing exciton lifetime, and we therefore expect a shorter *L*_D_. To account for differences between mutants with differing “dark” fluorescence lifetimes, we use the directly measured fluorescence lifetimes to correlate the extracted *L*_D_ values from the fifth-order responses in Figure 3. The data plotted in Figure 3 show a positive correlation between *L*_D_ and τ_F_. A exponential fit in Figure 3 is consistent with the exponential dependence of NPQ versus *L*_D_ purposed by Bennett et al.^25^ A fit of these data across quenching capacities and xanthophyll compositions gives an *L*_D_ = 38 nm at a τ_F_ of 1.3 ns.

**Figure 3:**
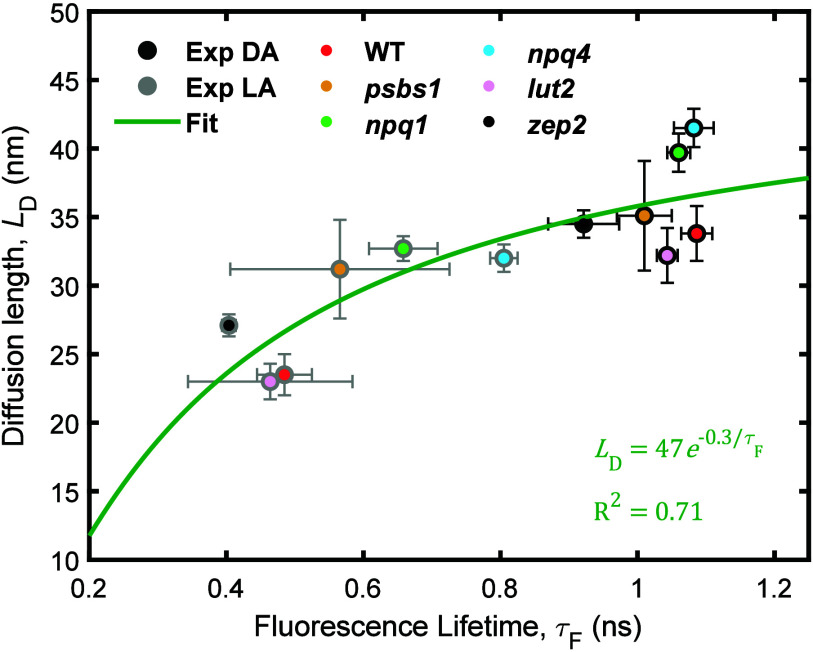
Correlation between *L*_D_ and τ_F_. The measured transient absorption data under dark (black edges) and light (gray edges) conditions are shown. The different inner color dots highlight the results of WT (red), *psbs1* (orange), *npq1* (green), *npq4* (blue), *lut2* (pink), and *zep2* (black) mutants. The green line is the fit using a single-exponential rising function for *L*_D_ vs τ_F_. The τ_F_ values for the DA data are averaged over the first minute of light exposure.

Comparison of the NPQ activity and exciton diffusion length among *npq4*, *psbs1*, and WT genotypes provides insight into the specific contribution of qE to exciton transport regulation. *npq4*, which lacks the PsbS protein and is deficient in qE, exhibits minimal NPQ induction and shows the longest *L*_D_ (32 nm) under light conditions. In contrast, WT shows substantial NPQ activation and a significant shorter *L*_D_ of 24 nm. *psbs1*, which retains partial PsbS expression, shows intermediate values in both τ_F_ and *L*_D_, suggesting a concentration-dependent relationship between PsbS-mediated qE activation and the reduction of exciton mobility. Notably, the additional reduction in *L*_D_ observed in WT compared to *npq4* under LA conditions indicates qE as the dominant component driving both enhanced NPQ activity and exciton mobility restriction.

The *L*_D_ value for *lut2* requires some discussion because of the likely differences in its thylakoid composition. The absence of lutein in *lut2* mutants leads to a looser antenna protein structure and a higher concentration of monomeric LHCII in thylakoid membranes than WT following weak solubilization *in vitro*.^17,32^ In addition, *npq4* mutants of *Arabidopsis thaliana* were observed to have distinct membrane organization, which may be associated with the lack of the PsbS protein,^44^ while no such differences were observed between the WT and *npq4* in *N. benthamiana.* ^17^ These structural changes may modify our assumption of similar exciton motion in these two mutants, influencing the effective *R* value and exciton diffusion by altering interprotein distances. For instance, although Figure 3 shows similar *L*_D_ values for *lut2* and WT, the actual *L*_D_ in *lut2* is likely underestimated due to the greater distance between loose LHCII proteins.

The fitted curve in Figure 3 suggests that the range of exciton motion in the thylakoid membrane (*L*_D_) when NPQ is absent is ∼38 nm. In this work, we implement the relative diffusion coefficient and exciton concentration in eq 2 for a more realistic scenario than in ref 17. The updated equation defined in this study results in *L*_D_ values of 31 ± 3 nm for the WT data in ref 17, in reasonable agreement with the WT value obtained in this work (34 nm). In excess light, the *L*_D_ value of WT decreases to 22–24 nm in both studies. We note that NPQ_τ_ values in isolated thylakoid membranes are always smaller than in intact leaves and, for NPQ_τ_ values larger than 2, *L*_D_ could be as small as 15 nm, corresponding to a τ_F_ of 0.24 ns.

To provide a qualitative context for these values, we consider a semicrystalline array of the C_4_S_4_M_2_ PSII megacomplexes, each measuring 32 nm × 29 nm,^45^ as an example of a compact energy transfer network, In such an array, an exciton can reach approximately 16 RCs when *L*_D_ = 38 nm. Reducing *L*_D_ to 15 nm lowers this number to 2–4 RCs within a single megacomplex and 0–3 RCs in neighboring complexes. Notably, reducing the system size does not diminish the influence of NPQ: *L*_D_ remains sensitive to NPQ activation even in smaller, isolated supercomplexes when quenchers are present.^25^ From a photoprotection perspective, a shorter *L*_D_ under high light conditions limits the number of excitons reaching reaction centers, thereby reducing the risk of overexcitation. In addition, a reduced exciton lifetime decreases the probability of intersystem crossing from singlet excited Chl to the triplet state, thereby lowering the potential for reactive oxygen species formation.

We conclude with some comments on potential complexities of the analysis presented here. The absolute values of our reported *L*_*D*_ values are highly dependent on the form of the diffusion model used. Several factors may contribute to variations in *L*_D_, including system size *V*, reaction radius *R*, exciton diffusion behavior, and the variation between biological replicates. The parameters *V* and *R* directly influence the evaluated diffusion coefficient, thereby affecting the final *L*_D_ values. While the absolute *L*_D_ values vary due to the model assumptions, the overall trend of *L*_D_ reduction with decreasing τ_F_ and thus increasing NPQ_τ_ should remain valid.

In previous work, we found that exciton motion in the C_2_S_2_ supercomplex exhibits subdiffusive behavior, with a subdiffusivity parameter α = 0.53, but the influence on *L*_D_ values was relatively minor (11% difference from normal diffusion).^46^ As the supercomplex size increases, we expect exciton motion to exhibit a larger α value, gradually approaching normal diffusion (α = 1). At present, distinguishing normal and anomalous diffusion in kinetic profile analysis is challenging because of the signal-to-noise ratio limitations in fifth-order nonlinear signals. In fits of the current data, the subdiffusive model yields an estimated subdiffusivity of close to 1 indicating that the analysis presented above provides a reasonable description of exciton motion in the thylakoid membrane of a land plant. In terms of relative changes, our results are consistent with a previous simulation, both showing a ∼40% reduction in *L*_D_ from its maximum when NPQ_τ_ reaches 2.^25^

In this study, potential structural reorganizations of the thylakoid membrane were not included in the diffusion model. However, under longer high-light illumination, dynamic changes such as LHCII aggregation and reorganization of PSII-LHCII supercomplex composition may significantly influence exciton diffusion behavior. These changes could alter the effective *V* and *R* parameters used in estimating *L*_D_, thereby impacting the interpretation of *L*_D_ under physiological conditions. The consistent decreases in *L*_D_ across genotypes observed here affirms the importance of reductions in exciton diffusion length on the rapid time scales at which reversible NPQ operates. Future work integrating membrane-structural dynamics with exciton transport models will be essential for a comprehensive understanding of different NPQ regulation mechanisms across varying time scales.

Transgenic *Nicotiana benthamiana* (accession *Nb-1*) lines were generated via *Agrobacterium*-mediated transformation by the Ralph M. Parsons Foundation Plant Transformation Facility at UC Davis (https://ptf.ucdavis.edu/). *N. benthamiana* plants were grown with a 10-h daylength in a south-facing greenhouse. Seeds were germinated directly on a mixture of four parts Sunshine Mix #1 (Sungro) and one part perlite, and fertilized with JR Peter’s Blue 20-20-20 fertilizer.

Five-week-old *N. benthamiana* plants were dark acclimated for 1 h. Whole leaves were then harvested wrapped in moist paper towels and aluminum foil, and stored overnight at 4 °C. Thylakoid membrane purification was performed in a dark cold room (4 °C), using a modified protocol based on Gilmore et al.^47^ Approximately 10 g of chilled leaf tissue was homogenized in a grinding buffer where 0.33 M sorbitol replaced dextrose, 0.2% l-ascorbic acid substituted for sodium ascorbate, and 10 mM EDTA was added; the final pH was adjusted to 8.2. After blending, the homogenate was filtered through four layers of Miracloth. To eliminate starch, the filtrate was subjected to a brief centrifugation at 1500*g* for 2 min. The resulting supernatant was carefully decanted into clean tubes and centrifuged again at 1500*g* for 10 min. The pellet obtained was gently resuspended in buffer A using a paintbrush to avoid disturbing any remaining starch granules. Chlorophyll concentration was determined by extracting the thylakoid samples in 80% acetone, following the method described by Porra et al.^48^ Prior to measurement, thylakoid suspensions were diluted to a final concentration of 75 μg Chl/mL in the reaction buffer (pH 8.0), which contained 30 mM L-ascorbic acid, 0.5 mM ATP, and 50 μM methyl viologen.

Exciton–exciton annihilation-free transient spectroscopy applied a pump–probe TA setup as described in the SI Methods. To obtain the high-order nonlinear TA signals up to seventh order (PP3, PP5, and PP7), we collected TA profiles with 6, 18, and 24 nJ pump intensity as a complete set of intensity-cycling measurements. Each set of measurements was conducted from the lowest to the highest pump intensity. The pump intensities were monitored by a power meter (PM100D, Thorlabs) before and after collecting a TA profile to ensure a consistent intensity during the measurement. The high order nonlinear signal is extracted using the following linear combination relationship developed by Malý et al.:^34^

$$\left[\begin{array}{l} \mathrm{PP}3\;I\\ \mathrm{PP}5\;I^{2}\\ \mathrm{PP}7\;I^{3} \end{array}\right]=\left[\begin{array}{lll} 2 & -2/3 & 1/4\\ -7/6 & 5/6 & -1/3\\ 1/6 & -1/6 & 1/12 \end{array}\right]\left[\begin{array}{l} \mathrm{PP}\left(6\mathrm{nJ}\right)\\ \mathrm{PP}\left(18\mathrm{nJ}\right)\\ \mathrm{PP}\left(24\mathrm{nJ}\right) \end{array}\right]$$ 4

The dark condition profiles were measured in a dark room with thylakoid samples dark-acclimated for more than 30 min. Following dark condition measurements, the high light condition profiles were measured after 15 min actinic light exposure at 1000 μmol photons m^–2^ s^–1^. Each set of intensity-cycling-based measurements was completed within 20 min, and the TA measurement of each thylakoid sample was completed within an hour to preserve the NPQ activity of the thylakoids.

## Supplementary Material



## Notes

Details of fluorescence lifetime snapshot measurements, transient absorption spectroscopy, and fitting functions used for PP3 and PP5 signals. Figures presenting PP3 and PP5 results for all genotypes and extracted high-order nonlinear signals for WT (PDF)

## References

[1] Yang S.-J., Arsenault E. A., Orcutt K., Iwai M., Yoneda Y., Fleming G. R. (2022). From antenna
to reaction center: Pathways of ultrafast
energy and charge transfer in photosystem II. Proc. Natl. Acad. Sci. U. S. A..

[2] Pagliano C., Saracco G., Barber J. (2013). Structural,
functional and auxiliary
proteins of photosystem II. Photosynth. Res..

[3] Van
Amerongen H., Croce R. (2013). Light harvesting in photosystem II. Photosynth. Res..

[4] Croce R., vanAmerongen H. (2020). Light harvesting in oxygenic photosynthesis:
Structural
biology meets spectroscopy. Science.

[5] Caffarri S., Broess K., Croce R., Van Amerongen H. (2011). Excitation
energy transfer and trapping in higher plant Photosystem II complexes
with different antenna sizes. Biophys. J..

[6] Demmig-Adams, B. ; Garab, G. ; Adams, W., III Non-Photochemical Quenching and Energy Dissipation in Plants, Algae and Cyanobacteria; Springer: Dordrecht, Netherlands, 2014.

[7] Müller P., Li X. P., Niyogi K. K. (2001). Non-photochemical quenching. A response
to excess light energy. Plant Physiol..

[8] Ruban A. V. (2016). Nonphotochemical
chlorophyll fluorescence quenching: Mechanism and effectiveness in
protecting plants from photodamage. Plant Physiol..

[9] Li X.-P. (2000). A pigment-binding protein
essential for regulation of photosynthetic
light harvesting. Nature.

[10] Erickson E., Wakao S., Niyogi K. K. (2015). Light stress
and photoprotection
in Chlamydomonas reinhardtii. Plant J..

[11] Demmig-Adams B., Polutchko S. K., Stewart J. J., Adams W. W. (2022). History of excess-light
exposure modulates extent and kinetics of fast-acting non-photochemical
energy dissipation. Plant Physiol. Reports.

[12] Jahns P., Latowski D., Strzalka K. (2009). Mechanism
and regulation of the violaxanthin
cycle: The role of antenna proteins and membrane lipids. Biochim. Biophys. Acta - Bioenerg..

[13] Demmig-Adams B., Adams W. (1996). Xanthophyll cycle and light stress
in nature: Uniform response to excess direct sunlight among higher
plant species. Planta.

[14] Park S. (2018). Chlorophyll-Carotenoid
Excitation Energy Transfer in High-Light-Exposed
Thylakoid Membranes Investigated by Snapshot Transient Absorption
Spectroscopy. J. Am. Chem. Soc..

[15] Horton P., Ruban A. V., Wentworth M. (2000). Allosteric
regulation of the light-harvesting
system of photosystem II. Philos. Trans. R.
Soc. B Biol. Sci..

[16] Short A. H., Fay T. P., Crisanto T., Hall J., Steen C. J., Niyogi K. K., Limmer D. T., Fleming G. R. (2022). Xanthophyll-cycle
based model of the rapid photoprotection of Nannochloropsis in response
to regular and irregular light/dark sequences. J. Chem. Phys..

[17] Lee T.-Y., Lam L., Patel-Tupper D., Roy P. P., Ma S. A., Lam H. E., Lucas-DeMott A., Karavolias N. G., Iwai M., Niyogi K. K., Fleming G. R. (2024). Chlorophyll
to zeaxanthin energy transfer in nonphotochemical
quenching: An exciton annihilation-free transient absorption study. Proc. Natl. Acad. Sci. U. S. A..

[18] Short A., Fay T. P., Crisanto T., Mangal R., Niyogi K. K., Limmer D. T., Fleming G. R. (2023). Kinetics
of the xanthophyll cycle
and its role in photoprotective memory and response. Nat. Commun..

[19] Johnson M. P., Pérez-Bueno M. L., Zia A., Horton P., Ruban A. V. (2009). The zeaxanthin-independent and zeaxanthin-dependent
qE components of nonphotochemical quenching involve common conformational
changes within the photosystem II antenna in Arabidopsis. Plant Physiol..

[20] Ruan M. (2023). Cryo-EM structures of LHCII in photo-active and photo-protecting
states reveal allosteric regulation of light harvesting and excess
energy dissipation. Nat. Plants.

[21] Michael
Gruber J., Chmeliov J., Krüger T. P. J., Valkunas L., Van Grondelle R. (2015). Singlet-triplet annihilation in single
LHCII complexes. Phys. Chem. Chem. Phys..

[22] Rutkauskas D., Chmeliov J., Johnson M., Ruban A., Valkunas L. (2012). Exciton annihilation
as a probe of the light-harvesting antenna transition into the photoprotective
mode. Chem. Phys..

[23] Barzda V. (2001). Singlet-singlet annihilation
kinetics in aggregates and trimers of
LHCII. Biophys. J..

[24] Swenberg C. E., Geacintov N. E., Breton J. (1978). Laser Pulse Excitation Studies of
the Fluorescence of Chloroplasts. Photochem.
Photobiol..

[25] Bennett D. I. G., Fleming G. R., Amarnath K. (2018). Energy-dependent quenching adjusts
the excitation diffusion length to regulate photosynthetic light harvesting. Proc. Natl. Acad. Sci. U. S. A..

[26] Li X. P., Müller-Moulé P., Gilmore A. M., Niyogi K. K. (2002). PsbS-dependent
enhancement of feedback de-excitation protects photosystem II from
photoinhibition. Proc. Natl. Acad. Sci. U. S.
A..

[27] Külheim C., Ågren J., Jansson S. (2002). Rapid regulation of light harvesting
and plant fitness in the field. Science.

[28] Sulli M., Dall’Osto L., Ferrante P., Guardini Z., Gomez R. L., Mini P., Demurtas O. C., Aprea G., Nicolia A., Bassi R., Giuliano G. (2023). Generation and physiological characterization
of genome-edited Nicotiana benthamiana plants containing zeaxanthin
as the only leaf xanthophyll. Planta.

[29] Rock C. D., Zeevaart J. A. D. (1991). The aba mutant
of Arabidopsis thaliana is impaired
in epoxy-carotenoid biosynthesis. Proc. Natl.
Acad. Sci. U. S. A..

[30] Niyogi K. K., Grossman A. R., Björkman O. (1998). Arabidopsis mutants define a central
role for the xanthophyll cycle in the regulation of photosynthetic
energy conversion. Plant Cell.

[31] Pogson B., McDonald K. A., Truong M., Britton G., DellaPenna D. (1996). Arabidopsis
carotenoid mutants demonstrate that lutein is not essential for photosynthesis
in higher plants. Plant Cell.

[32] Dall'Osto L., Lico C., Alric J., Giuliano G., Havaux M., Bassi R. (2006). Lutein is needed for
efficient chlorophyll triplet quenching in the
major LHCII antenna complex of higher plants and effective photoprotection
in vivo under strong light. BMC Plant Biol..

[33] Patel-Tupper D., Kelikian A., Leipertz A., Maryn N., Tjahjadi M., Karavolias N. G., Cho M.-J., Niyogi K. K. (2024). Multiplexed CRISPR-Cas9
mutagenesis of rice PSBS1 noncoding sequences for transgene-free overexpression. Sci. Adv..

[34] Malý P. (2023). Separating single- from multi-particle dynamics in nonlinear spectroscopy. Nature.

[35] Blankenship, R. E. Molecular Mechanisms of Photosynthesis, 3rd.; Wiley-Blackwell: Chichester, West Sussex, UK, 2008.

[36] Sylak-Glassman E. J. (2014). Distinct roles of the photosystem II protein PsbS and zeaxanthin
in the regulation of light harvesting in plants revealed by fluorescence
lifetime snapshots. Proc. Natl. Acad. Sci. U.
S. A..

[37] Sylak-Glassman E. J., Zaks J., Amarnath K., Leuenberger M., Fleming G. R. (2016). Characterizing non-photochemical quenching in leaves
through fluorescence lifetime snapshots. Photosynth.
Res..

[38] Lam L. (2024). Resolving
an unconventional non-photochemical quenching signature
at the light-to-dark transition. bioRxiv.

[39] Horton, P. Developments in Research on Non-Photochemical Fluorescence Quenching: Emergence of Key Ideas, Theories and Experimental Approaches. In Advances in Photosynthesis and Respiration Non-Photochemical Quenching and Energy Dissipation in Plants, Algae and Cyanobacteria; Demmig-Adams, B. , Garab, G. , Adams, W., III ; Govindjee ; Springer Netherlands: Dordrecht, 2014; pp 73–95.

[40] Ünnep R. (2020). Thylakoid membrane reorganizations revealed by small-angle neutron
scattering of Monstera deliciosa leaves associated with non-photochemical
quenching. Open Biol..

[41] Kumar S. (2023). Exciton annihilation in molecular aggregates suppressed through qu
antum interference. Nat. Chem..

[42] Torney D. C., McConnell H. M. (1983). Diffusion-Limited
Reaction Rate Theory for Two-Dimensional
Systems. Proc. R. Soc. London A. Math. Phys.
Sci..

[43] Yang S., Wales D. J., Woods E. J., Fleming G. R. (2024). Design principles
for energy transfer in the photosystem II supercomplex from kinetic
transition networks. Nat. Commun..

[44] Kiss A. Z., Ruban A. V., Horton P. (2008). The PsbS protein
controls the organization
of the photosystem II antenna in higher plant thylakoid membranes. J. Biol. Chem..

[45] Shan J., Niedzwiedzki D. M., Tomar R. S., Liu Z., Liu H. (2024). Architecture
and functional regulation of a plant PSII-LHCII megacomplex. Sci. Adv..

[46] Zhang K., Lee T.-Y., Yang S.-J., Bhagde T., Iwai M., Fleming G. R. (2025). Probing exciton diffusion dynamics in photosynthetic
supercomplexes via exciton–exciton annihilation. J. Chem. Phys..

[47] Gilmore A.
M., Shinkarev V. P., Hazlett T. L., Govindjee (1998). Quantitative analysis of the effects
of intrathylakoid pH and xanthophyll cycle pigments on chlorophyll
a fluorescence lifetime distributions and intensity in thylakoids. Biochemistry.

[48] Porra R. J., Thompson W. A., Kriedemann P. E. (1989). Determination of accurate extinction
coefficients and simultaneous equations for assaying chlorophylls
a and b extracted with four different solvents: verification of the
concentration of chlorophyll standards by atomic absorption spectroscopy. Biochim. Biophys. Acta - Bioenerg..

