# Supplementary material for: Reduction of Exciton Diffusion Length with Genetically Tuned Non-Photochemical Quenching in Plant Thylakoid Membranes
Source: J Phys Chem Lett. 2025 Jul 14;16(29):7352–8. doi: 10.1021/acs.jpclett.5c01473 (PMC12302212; doi:10.1021/acs.jpclett.5c01473)
Supplement: Supplementary file 1 [file jz5c01473_si_001.pdf]

Reduction of Exciton Diffusion Length with Genetically Tuned Non-Photochemical Quenching in Plant Thylakoid Membranes

Supporting Information

Tsung-Yen Lee[1,2], Lam Lam[1,3], Dhruv Patel-Tupper[4,5], Henry E. Lam[1], Krishna K. Niyogi[2,4,5,6], and Graham R. Fleming[1,2,7]*

[1]Department of Chemistry, University of California, Berkeley, CA 94720, United States.
[2]Molecular Biophysics and Integrated Bioimaging Division, Lawrence Berkeley National Laboratory, Berkeley, CA 94720, United States.
[3]Graduate Group in Biophysics, University of California, Berkeley, CA 94720, United States.
[4]Department of Plant and Microbial Biology, University of California, Berkeley, CA 94720, United States.
[5]Howard Hughes Medical Institute, University of California, Berkeley, CA 94720, United States.
[6]Innovative Genomics Institute, University of California, Berkeley, CA 94720, United States.
[7]Kavli Energy Nanoscience Institute at Berkeley, Berkeley, CA 94720, United States.

Corresponding author: Graham R. Fleming (Email: fleming@berkeley.edu)

Supporting Methods

Fluorescence Lifetime Snapshot Measurements

To measure Chl fluorescence lifetimes of the thylakoid samples, we employed time-correlated single photon counting (TCSPC), following a previously established protocol. [1] The excitation source was a Ti:sapphire oscillator (Coherent Mira900f, 76 MHz) that produced ~808 nm pulses. These pulses were frequency-doubled to ~404 nm via a beta barium borate (BBO) crystal to excite the Soret band of Chl a. A beam splitter directed part of the beam to a photodiode (Becker-Hickl PHD-400) for generating SYNC signals, while the remaining beam was aimed at the sample cuvette at an incidence angle of ~70° to the cuvette surface with the power set to 1.0 mW, sufficient to saturate the PSII reaction centers. The beam diameter at the sample position was ~600 μm. Throughout the measurements, samples were subjected to a programmed actinic light regime using a Leica KL1500 LCD source, alternating between high-light (1000 μmol photons m^{-2} s^{-1}) and darkness in a 15-5-5-5 minute sequence. Chl a Qy band fluorescence emission was selected at 680 nm by a monochromator (HORIBA Jobin-Yvon; H-20) and detected by a microchannel plate (MCP)-photomultiplier tube (PMT) detector (Hamamatsu R3809U MCP-PMT). The excitation, actinic light and detection were controlled by their corresponding shutters using a custom LabVIEW program. Each snapshot was taken at intervals of 30 s. Each fluorescence decay profile over 10 ns was fit to a bi-exponential decay function, using a wider fitting window than the TA measurement (~200 ps), which may account for the differences in extracted lifetimes between the two methods. The amplitude-weighted average lifetime was calculated as:

$$\tau_{\mathrm{F}} = \frac{\sum_i \mathrm{A}_i \, \tau_{\mathrm{F},i}}{\sum_i \mathrm{A}_i} \qquad \text{(S1)}$$

where A_i and $\tau_{\mathrm{F},i}$ are the amplitudes and fluorescence lifetimes of the ith fitting component, respectively. The NPQ capacity is defined by $\mathrm{NPQ}_{\tau}(T) = \frac{\tau_{\mathrm{F},dark} - \tau_{\mathrm{F},light}(T)}{\tau_{\mathrm{F},dark}}$ as described in the main text.

Transient Absorption Spectroscopy

The pump-probe TA system used a regenerative amplifier (RegA 9050, Coherent) seeded by Ti/sapphire Laser (Vitara-T, Coherent) to generate mode-locked 800 nm laser pulses at 250 kHz repetition rate. The pulse was modulated by an external stretcher/compressor and then split into pump and probe beam path by a beam splitter. The pump pulse was centered at 674 nm (FWHM 35 nm) with an optical parametric amplifier (OPA, Coherent) and compressed by prisms to a FWHM of an autocorrelation trace of ~49 fs. The pump intensity was adjusted between 0.8 to 32 nJ by a neutral-density filter wheel. For the probe beam path, a visible continuum was generated by a 1 mm sapphire crystal and filtered by a 700-nm short-pass filter. The pump and probe pulses passed through a 0.5 mm thick cuvette and were overlapped on a sample at the magic-angle (54.7°) polarization. The pump-probe cross-correlation time was ~100 fs at 680 nm, and the diameter of pump and probe pulses at the sample position was 160 and 80 μm, respectively. To prevent continuous excitation of a single spot, the cuvette was vibrated at 7 Hz in a direction perpendicular to the probe beam path. After passing through the sample, the probe beam was filtered by a polarizer to remove scattered pump light. A monochromator (SpectraPro 300i, Acton Research Corp.) was used to select probe wavelength (680 nm for chlorophyll ground state bleach). The exit pulses were collected by a diode detector (DET10A, Thorlabs), generating analog signal input to a lock-in amplifier (SR830, Stanford Research) which synchronized the pump-probe signals with a chopper positioned in the pump beam path.

Probing Exciton Diffusion with EEA Dynamics

The extracted PP3 and PP5 signal are fitted using the corresponding response function. The PP3 signal represent the signal exciton dynamics, described by a two-exponential decay function**:**

$$\mathrm{PP3}(t) = \sum_{i} \mathrm{A}_i e^{t/\tau_i} \qquad \text{(S2)}$$

where A are pre-exponential term, and τ_i is the annihilation rate constant. The averaged decay time is calculated by the amplitude-weight averaged method equivalent to equation (S1).

The PP5 signal involve both the single exciton decay and EEA dynamics. According to Malý's excitonic model[2], the response function of PP5 for a system with numerous pigments can be written as follows:

$$\mathrm{PP5}(t) = \mathrm{A}\left(1 - e^{\frac{-t}{\tau_{\mathrm{A}}}}\right) * \mathrm{PP3}(t) \quad \text{(S3)}$$

where A is a pre-exponential constant. $\mathrm{PP3}(t)$ depicts the single-particle dynamics and is substituted by the fitted function of the PP3 profile. The τ_{A} is a rise time constant corresponding to the EEA dynamics. The τ_{A} values are input to Eq (2) for evaluating the diffusion coefficient. Additionally, the reaction radius (*R*) is set as 2 nm, representing the mean distance between Chl energy transfer clusters. The system size (*V*) is calculated based on a cylindrical model with 4 nm thylakoid membrane thickness and a radius of 15 nm, representing half of the averaged exciton distance under our TA measurement setup.

For the sub-diffusion model, we fitted PP5 profiles with a fractalized method to describe the anomalous diffusive behavior [3], yielding

$$\mathrm{PP5}(t) = -\mathrm{A}\left(1 - e^{\frac{-t^{\alpha}}{\alpha\,\tau_{\mathrm{A0}}}}\right) * \mathrm{PP3}(t) \quad \text{(S4)}$$

where τ_{A0} is a parametric constant. α represents sub-diffusivity, where $\alpha = 1$ indicates a normal diffusive behavior and $\alpha < 1$ is a sub-diffusive process.

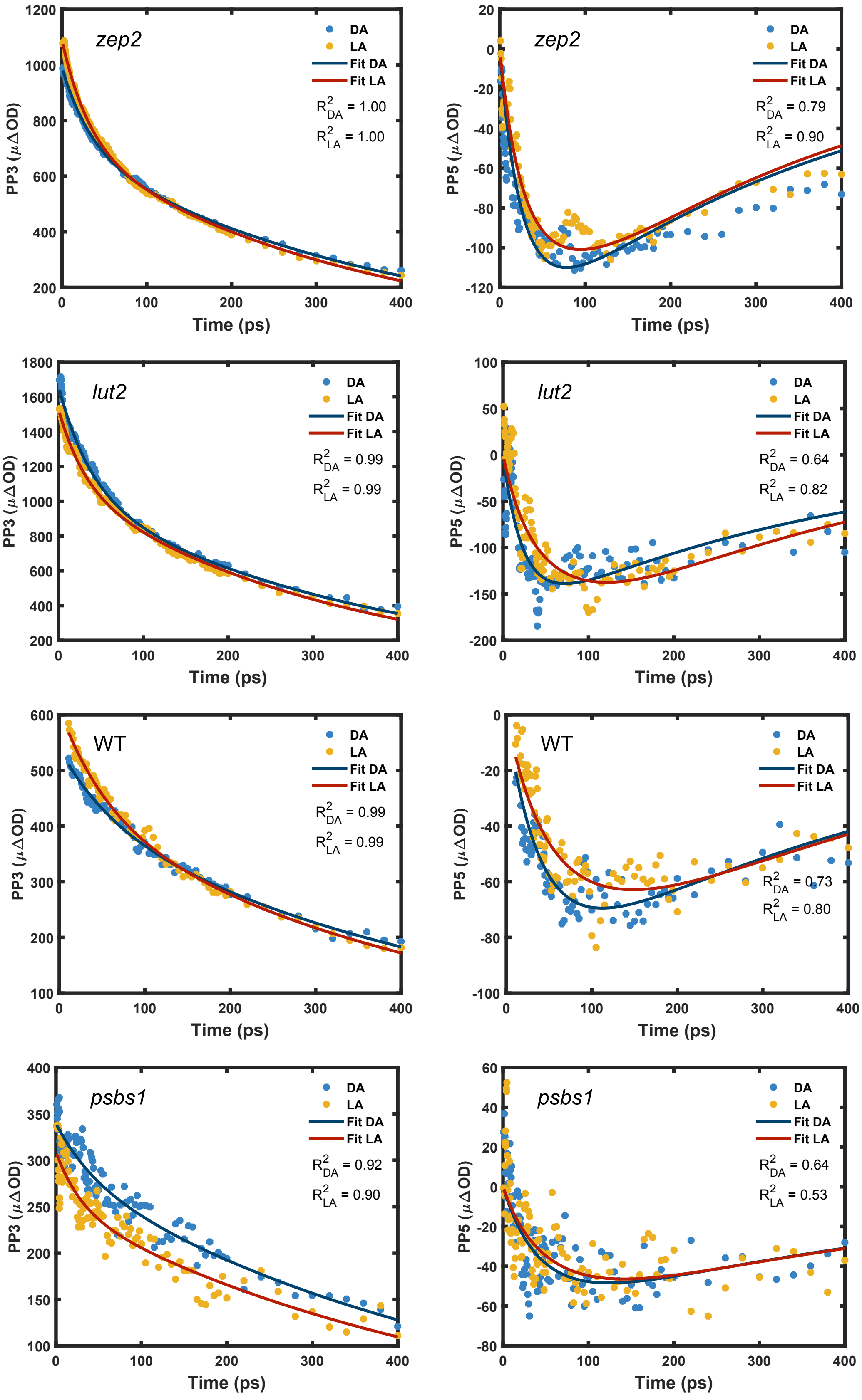
zep2
DA
LA
Fit DA
Fit LA
R²DA = 1.00
R²LA = 1.00
PP3 (μΔOD)
Time (ps)
zep2
R²DA = 0.79
R²LA = 0.90
PP5 (μΔOD)
lut2
R²DA = 0.99
R²LA = 0.99
lut2
R²DA = 0.64
R²LA = 0.82
WT
R²DA = 0.99
R²LA = 0.99
WT
R²DA = 0.73
R²LA = 0.80
psbs1
R²DA = 0.92
R²LA = 0.90
psbs1
R²DA = 0.64
R²LA = 0.53

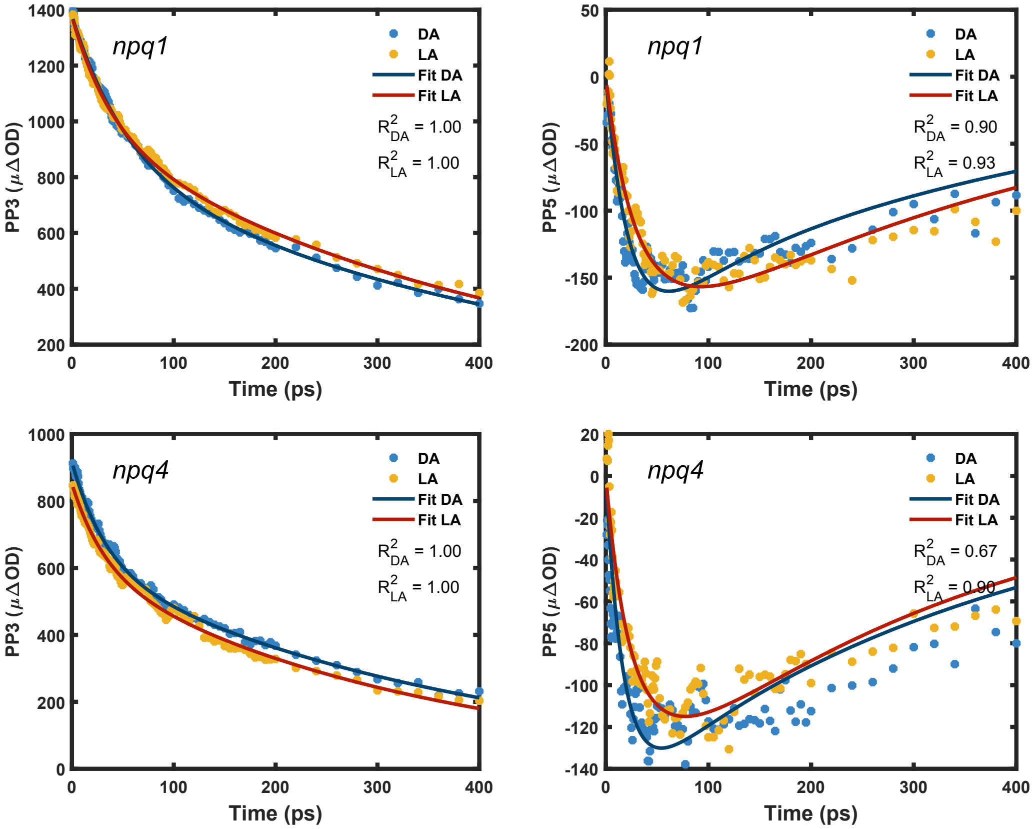

Figure S1 The extracted (left) PP3, (right) PP5 results, and corresponding fitting results for all genotypes in *N. benthamiana* thylakoid membranes.

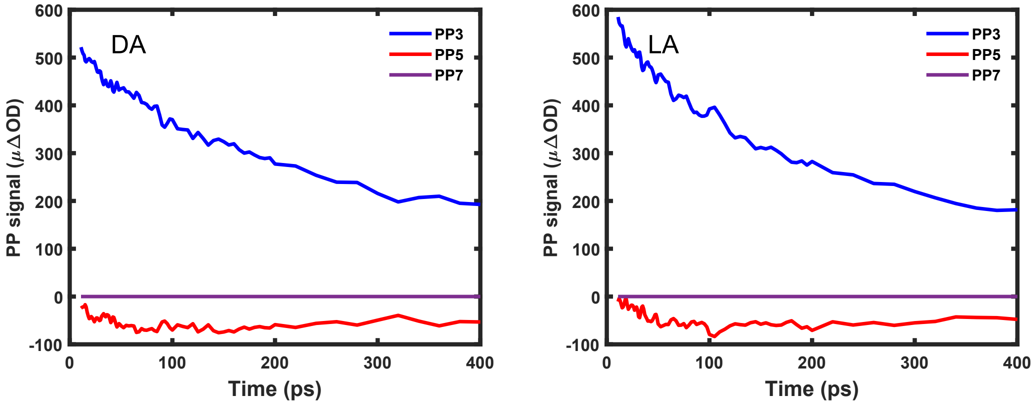

Figure S2 The extracted PP3, PP5, and PP7 results for WT *N. benthamiana* thylakoid membranes under (left) DA and (right) LA conditions. The amplitude of 7th-order nonlinear signal is close to zero and below the detection limit, indicating that contributions from higher-order signals can be neglected.

References

[1] Steen, C. J., Morris, J. M., Short, A. H., Niyogi, K. K. &Fleming, G. R. Complex Roles of PsbS and Xanthophylls in the Regulation of Nonphotochemical Quenching in Arabidopsis thaliana under Fluctuating Light. *J. Phys. Chem. B* **124**, 10311–10325 (2020).

[2] Malý, P. *et al.* Separating single- from multi-particle dynamics in nonlinear spectroscopy. *Nature* **616**, 280–287 (2023).

[3] Bunde, A. &Havlin, S. *Fractals And Disordered Systems*. (Springer Berlin, Heidelberg, 1991).
